# Theta-gamma coupling binds visual perceptual features in an associative memory task

**DOI:** 10.1038/s41598-018-35812-7

**Published:** 2018-12-06

**Authors:** Moritz Köster, Holger Finger, Sebastian Graetz, Maren Kater, Thomas Gruber

**Affiliations:** 1 Institute of Psychology, Osnabrück University, 49074 Osnabrück, Germany; 2 Institute of Psychology, Freie Universität Berlin, 14195 Berlin, Germany; 3 Institute of Cognitive Science, Osnabrück University, 49069 Osnabrück, Germany

## Abstract

It is an integral function of the human brain to sample novel information from the environment and to integrate them into existing representations. Recent evidence suggests a specific role for the theta rhythm (4–8 Hz) in mnemonic processes and the coupling between the theta and the gamma rhythm (40–120 Hz) in ordering and binding perceptual features during encoding. Furthermore, decreases in the alpha rhythm (8–12 Hz) are assumed to gate perceptual information processes in semantic networks. In the present study, we used an associative memory task (object-color combinations) with pictures versus words as stimuli (high versus low visual information) to separate associative memory from visual perceptual processes during memory formation. We found increased theta power for later remembered versus later forgotten items (independent of the color judgement) and an increase in phase-amplitude coupling between frontal theta and fronto-temporal gamma oscillations, specific for the formation of picture-color associations. Furthermore, parietal alpha suppression and gamma power were higher for pictures compared to words. These findings support the idea of a theta-gamma code in binding visual perceptual features during encoding. Furthermore, alpha suppression likely reflects perceptual gating processes in semantic networks and is insensitive to mnemonic and associative binding processes. Gamma oscillations may promote visual perceptual information in visual cortical networks, which is integrated into existing representations by prefrontal control processes, working at a theta pace.

## Introduction

To retain a coherent internal representation of the outer world, the wake human brain constantly samples and integrates information from the environment^1,2^. The integration of novel information relies on the formation of new associations between perceptual information, to form a coherent spatio-temporal representation of the environment^3^. This way, mnemonic processes are inextricably linked to perceptual processes^4^, the activation of semantic neuronal networks and the integration of novel sensory information. Specifically, Cowan conceptualizes perceptual processes as the activation of existing neuronal representations (the long-term memory storage), which may then be bound into memories (forming new associations in cortical semantic networks).

On a neuronal time scale, the activity within and across nerve cell populations is coordinated and integrated by the rhythmic synchronization of neuronal activity^5–7^ and it is assumed that neuronal rhythms are key mechanisms, which facilitate perceptual processes^8^ and enable the formation of new memories^9^. To observe memory formation processes in the human brain, in subsequent memory paradigms, the neuronal activity during encoding is contrasted between later remembered and later forgotten items^10^. Previous electro- and magnetoencephalography (EEG and MEG) studies have established that the formation of novel memory traces is closely associated with neuronal oscillatory activity in the theta (3–8 Hz), alpha (8–12 Hz), and gamma (40–120 Hz) frequency in the human brain^11–13^. Specifically, successful encoding in memory tasks using visual stimuli was marked by increases in theta and gamma power and a decrease in alpha power^11,13^.

Although visual perceptual and mnemonic mechanisms are inextricably linked^4^, oscillatory dynamics at different frequency ranges are assumed to index distinct functional mechanisms during memory formation. Theta oscillations facilitate associative binding^14^, play a functional role in integrating of novel perceptual information into lasting memory traces^15,16^, and increase with the intentional encoding of visual perceptual features^17^. Gamma oscillations in the visual cortex reflect the promotion of perceptual signals along the visual hierarchy^18^, and the sustained activation of recent perceptual inputs in working memory^19,20^. Decreases in alpha activity are assumed to facilitate visual cortical processes during memory formation^11,21,22^. Specifically, the alpha rhythm was decreased for the memory formation in semantic encoding tasks^17,22^ and was insensitive to the intentional encoding of perceptual features^17^. Alpha oscillations are assumed to inhibit neuronal activity in task-irrelevant regions, while routing perceptual processes to task-relevant regions^23^. Thus, reduced alpha oscillation may reflect the activation of semantic cortical networks^24^ and thereby form a functional architecture on which binding mechanisms operate^23^.

Recently, a specific role for mnemonic processing in the human brain has been ascribed to theta-gamma coupling processes. The frontal theta rhythm is posited to reflect an executive mnemonic mechanism that acts on perceptual information, reflected in gamma activity^11,25^. This is, theta-gamma coupling reflects the maintenance, ordering and binding of perceptual information within neuronal networks, forming a theta-gamma neuronal code^26^. This is substantiated by theta-gamma phase-amplitude coupling (PAC) pattern in the human neocortex^27^ and an increase in theta-gamma PAC in neocortical and medio-temporal networks accompanying successful episodic encoding^11,28,29^ and working memory processes^19^. A recent study using visual brain stimulation at an individual theta rhythm (versus an individual alpha rhythm) provides first functional evidence for the theta-gamma neuronal code in memory formation processes^16^. The theta-gamma code is assumed to support long-term potentiation processes in the hippocampus^30^, the core system of human associative memory formation^3^. However, despite a clear theoretical dissociation between the oscillatory dynamics that underpin memory encoding^31^, mnemonic and visual perceptual processes have not been separated experimentally, thus far.

In the present encephalographic (EEG) study, we aimed to dissociate the neuronal oscillatory activity for associative binding and visual perceptual processes during memory formation. To pinpoint associative memory processes, we used a subsequent memory design with an associative encoding and retrieval task. Furthermore, to scrutinize perceptual processes in the visual modality, we used picture and word stimuli (i.e., high and low visual information). Our analysis focused on oscillatory dynamics at individual theta, alpha and gamma frequencies, as well as theta-gamma PAC dynamics, and alpha-gamma PAC as a control analysis. We hypothesized that theta power and theta-gamma coupling reflects the formation of associative memories (i.e., a subsequent memory effect), while alpha suppression and gamma oscillations as such would primarily reflect visual perceptual processes (i.e., being pronounced for pictures versus words).

## Methods

### Subjects

The final sample consisted of 26 subjects (18 female, *M*_age_ = 20.0 years, *SD*_age_ = 2.0 years). Two additional participants were not included in the analysis due to incomplete data assessments (*n = *2). The experimental procedure was conducted in accordance with the World Medical Association’s Declaration of Helsinki (59th WMA general assembly, Seoul, 2008) and informed written consent was obtained from each participant. According to the regulations on freedom of research in the German Constitution (§ 5 (3)), and the German University Law (§ 22) this study did not require a separate vote by a local Institutional Review Board. All data will be made available on reasonable request.

### Stimuli and Procedure

**Figure 1:**
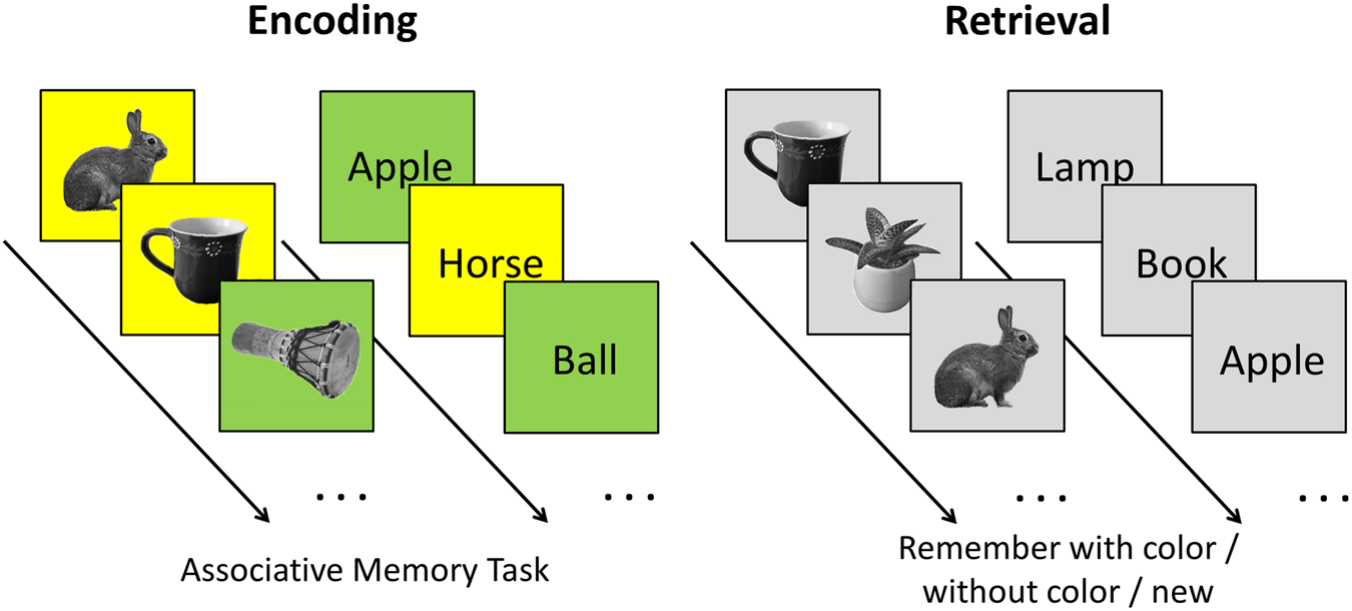
The experimental procedure and the stimuli used for the associative memory task. During encoding, subjects had to from associations between pictures (high visual information) or words (low visual information) and the background color (yellow or green). During retrieval, subjects were asked to recognize objects and to recall the associated color, among distractor items. Stimulus presentation was identical for encoding and retrieval: each trial started with a blank screen (s), followed by a fixation point (variable duration of 0.5–1.0 s), and the presentation of a target stimulus (s). The trial terminated with a question mark that remained until a response was given.

The pictorial stimuli were 400 pictures of objects (e.g. plants, animals, clothes, tools), taken from a standard picture library (Hemera Photo Objects; the exemplary stimuli shown in Fig. 1 were taken by the first author). Pictures were gray-scaled and presented at a visual angle of 6.2 × 6.2°. The words were 400 German nouns labeling familiar objects (with a maximum of 7 letters and 3 syllables).

The experimental procedure is depicted in Fig. 1. During encoding, participants were confronted with 300 pictures and 300 words, superimposed on a yellow or a green square. The associative memory task was to form object-color associations. Subjects were told the example of “a flower on green grass” as a potential association for the word or the picture of a flower presented on a green background. We avoided any overlaps between the objects presented as words and pictures (i.e., if a dog was presented as a picture, ‘dog’ was not used as a word). During retrieval, the pictures or words of the objects from encoding were intermixed with 50 new object stimuli of the respective category and presented on a gray background. For each picture, participants had to indicate, whether they retrieved the picture with the associated color (old, green or old, yellow), retrieved the picture without a color (old, no color) or did not see this picture before (new).

The stimulus presentation was identical during encoding and retrieval: each trial started with a blank screen (1 s), followed by a fixation point (variable duration of 0.5–1.0 s), and the presentation of a target stimulus (2 s). The trial terminated with a question mark, which remained on the screen until a response was given. Response keys were pressed with different fingers of the right hand. The procedure was demonstrated in 10 training trials prior to each phase of the actual experiment. We used a blocked design, presenting half of the pictorial stimuli (150, A), and half of the words (150, B), before presenting the other half of both categories (A, B). The order of the stimulus category presented first (pictures or words) was counterbalanced across participants resulting in an ABAB or BABA block logic. Object-color allocations (yellow or green) were randomized for each participant.

The present paper is a companion paper of a sleep study^32^. Thus, the memory for half of the stimuli was tests before, the other half after a three hour interval (i.e., after a sleep or wake interval of the sleep study). However, all stimuli were encoded at the same time, representing the data analyzed here.

### EEG Recording and Analyses

The EEG was recorded from 128 active electrodes using a BioSemi Active-Two amplifier system (BioSemi, Amsterdam) at a sampling rate of 512 Hz in a shielded room. A horizontal and vertical EOG was applied to monitor eye movements and blinks. Two additional electrodes (CMS: Common Mode Sense and DRL: Driven Right Leg; *cf*. www.biosemi.com/faq/cms&drl.htm) served as reference and ground.

Prior to the analyses, continuous EEG data was high-pass filtered at 0.5 Hz and eye-blinks and muscle artifacts were detected using an independent component procedure and removed after visual inspection^33^. EEG data was then segmented into epochs from −1000 ms to 3000 ms with regard to the stimulus onset. Further artifacts and noisy trials were removed by the means of statistical correction of artifacts in dense array studies (SCADS^34^), like in former studies^11,35^. At least 18 trials remained for each condition of each participant and thus all remaining participants were included in the final analyses. Furthermore, we applied a correction of saccade-related transient potentials^36^, used in several previous publications^25,32,36^ to remove miniature eye-movement artifacts^37^. Due to artifact correction procedures approximately 10% of the original trials were removed. Throughout further analysis, an average reference was used. To obtain the spectral power over time, the trial data was convoluted using Morlet’s wavelets with seven cycles^38^ at a resolution of 0.5 Hz.

#### Theta, alpha and gamma spectral power

To account for the variability in frequency bands across individuals^21^, we identified the peak theta, alpha and gamma frequencies, individually for each subject, based on the mean spectral activity across all encoding conditions^11,25,32^. This resulted in (Mean ± *SD*): 3.8 Hz ± 0.8 Hz theta frequencies, 9.8 Hz ± 2.2 Hz alpha frequencies and 57.3 Hz ± 10.2 Hz gamma frequencies. Event-related spectral changes at individual peak frequencies were then calculated as the relative signal change of the post stimulus spectral activity, relative to a −500 to −100 ms pre-stimulus baseline, in percent. For all topographical analyses, the relative signal change values were averaged over the time of stimulus presentation (500 to 2000 ms), excluding early evoked responses^11,13,25^ (0 to 500 ms), for all comparisons.

**Figure 2:**
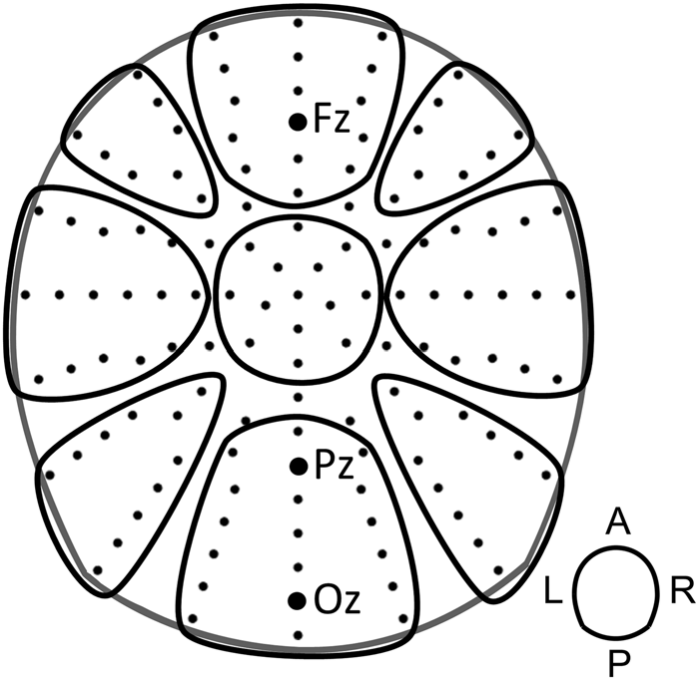
Electrode configuration and the clusters used for the statistical analyses. Electrodes were grouped along two dimensions, divided into 3 laterality (left, medial, right) times 3 caudality (frontal, central, posterior) clusters.

First, to analyze the overall spectral changes for theta, alpha and gamma frequencies in the encoding phase, the relative signal change values were tested against zero, using a cluster mass permutation *t*-test (two-sided, dependent samples)^39^. Second, to test the differences in theta, alpha and gamma activity between encoding conditions and stimulus type, we entered the relative signal change values of all trials into a repeated measures ANOVA with the factors 3 Response (SF, subsequently forgotten; SR, subsequently remembered with correct, or without color judgement), 2 Stimulus (picture, word), 9 Cluster (i.e., a 3 caudality × 3 laterality clustering; see Fig. 2). This strategy was chosen as a conservative method to include the oscillatory activity across the whole scalp. Huynh-Feldt corrected p-values are reported for all main effects and interactions. All effects not mentioned in the result section were non-significant, at the level of *p* > 0.10.

#### Theta-gamma phase-amplitude coupling

Phase-amplitude coupling (PAC) between the phase of both low frequencies, theta and alpha, and the gamma amplitude, were calculated using the modulation index^40^ (MI). Specifically, the individual theta and alpha oscillatory activity were filtered +/−1 Hz around the individual peak frequency, across fronto-central electrode clusters for theta and at parietal electrode clusters for alpha. Specifically, we choose fronto-central electrodes for theta and parietal electrodes for alpha because these are the regions where these rhythms are most pronounced^11,25^ and their phase can thus be most reliably determined. The gamma signal was filtered between 50–80 Hz, a frequency range adapted from previous studies^11,16,25^, at each of the 128 electrodes. The MI was then calculated between the theta phase of fronto-central electrodes^11,25^ and the gamma amplitude, separately for each electrode, for the 500–2000 ms time window. To avoid any bias due to different trial numbers, the trial number of each condition were matched to the lowest number of trials within the test conditions (SR [color], SR [no color], SF), for each participant.

We then calculated a three factorial repeated measures ANOVA equal to the one used in spectral power analysis (3 Response × 2 Stimulus × 9 Cluster). Because theta-gamma coupling during memory formation has formerly been found to be a local effect^11,19^, we furthermore conducted a cluster permutation t-test (two-sided, dependent samples; Maris and Oostenveld, 2007). This is, we tested for significant electrode clusters in all SME contrasts of interest (SME [color] and SME [no color] for pictures and words) against randomly occurring clusters in all contrasts of interest, when conditions were shuffled. Specifically, the cluster calculation sums up all t-values of neighboring electrodes with a p-value < 0.05. To obtain a valid distribution for the calculation of significance 10000 Monte Carlo iterations with randomly assigned conditions, were calculated for each contrast. For the final statistics, the distributions of all SME contrasts were combined into one single distribution, to estimate the significance of the summed cluster t-values (combined cluster permutation test; cf. Köster *et al*., 2017). Noteworthy, by combining the permutation distributions of all SME differences, the significance test takes into account the overall differences in neuronal activity across all conditions.

## Results

### Behavioral Results

Response rates for old and new items are displayed in Table 1. We used *d’* to quantify participants item memory (based on hits and false alarms, independent of the color judgement) and their associative memory performance (based on correct color judgements as hits and incorrect color judgements false alarms), separately for pictures and words^32^. Note that both measures reflect either only item memory or only item-color associations and are independent.

**Table 1:** Response rates in percent.

Picture	Response	Picture	Word	*t*(25)	*p*
Old	Hit (color)	37.6 (0.15)	40.2 (0.13)	−1.47	0.154
	Hit (false color)	12.1 (0.05)	12.1 (0.06)	−0.2	0.987
	Hit (no color)	27.8 (0.10)	25.5 (0.11)	1.88	0.073
	Miss	22.5 (0.09)	22.2 (0.12)	0.15	0.884
New	False alarm (color)	9.4 (0.06)	13.8 (0.10)	−2.58	0.016
	False alarm (no color)	11.2 (0.08)	22.1 (0.13)	−5.68	<0.001
	Correct rejection	79.5 (0.14)	64.1 (0.18)	5.93	<0.001

Mean ± SD response rates are displayed in percent of old and new items, respectively. −values denote differences between pictures and words (results of pairwise comparisons, two-sided).

Overall, we found higher item memory performance for pictures (*d’* = 1.70, *SD* = 0.55), compared to words (*d’* = 1.23, *SD* = 0.45), *t*(25) = 4.23, *p* < 0.001, due to higher false alarm rates for word stimuli, see Table 1. There were no differences in associative memory performance between words (*d’* = 0.98, *SD* = 0.44) and pictures (*d’* = 0.88, *SD* = 0.45), *t*(25) = −1.49, *p = *0.149.

### EEG Results

#### Spectral power

**Figure 3:**
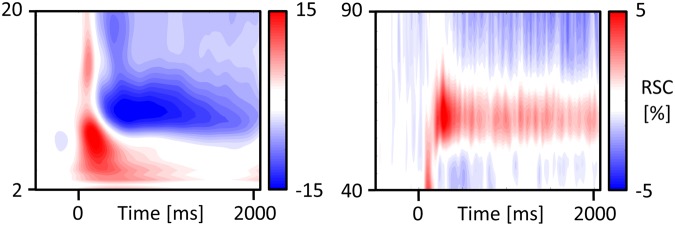
Time-frequency plots illustrate the relative signal changes (RSC) upon stimulus onset across all subjects and encoding conditions included in the analyses (linear y-axis, indicating Hz). RSC were averaged across frontal and posterior electrodes for low frequencies and across posterior electrodes for high frequencies.

**Figure 4:**
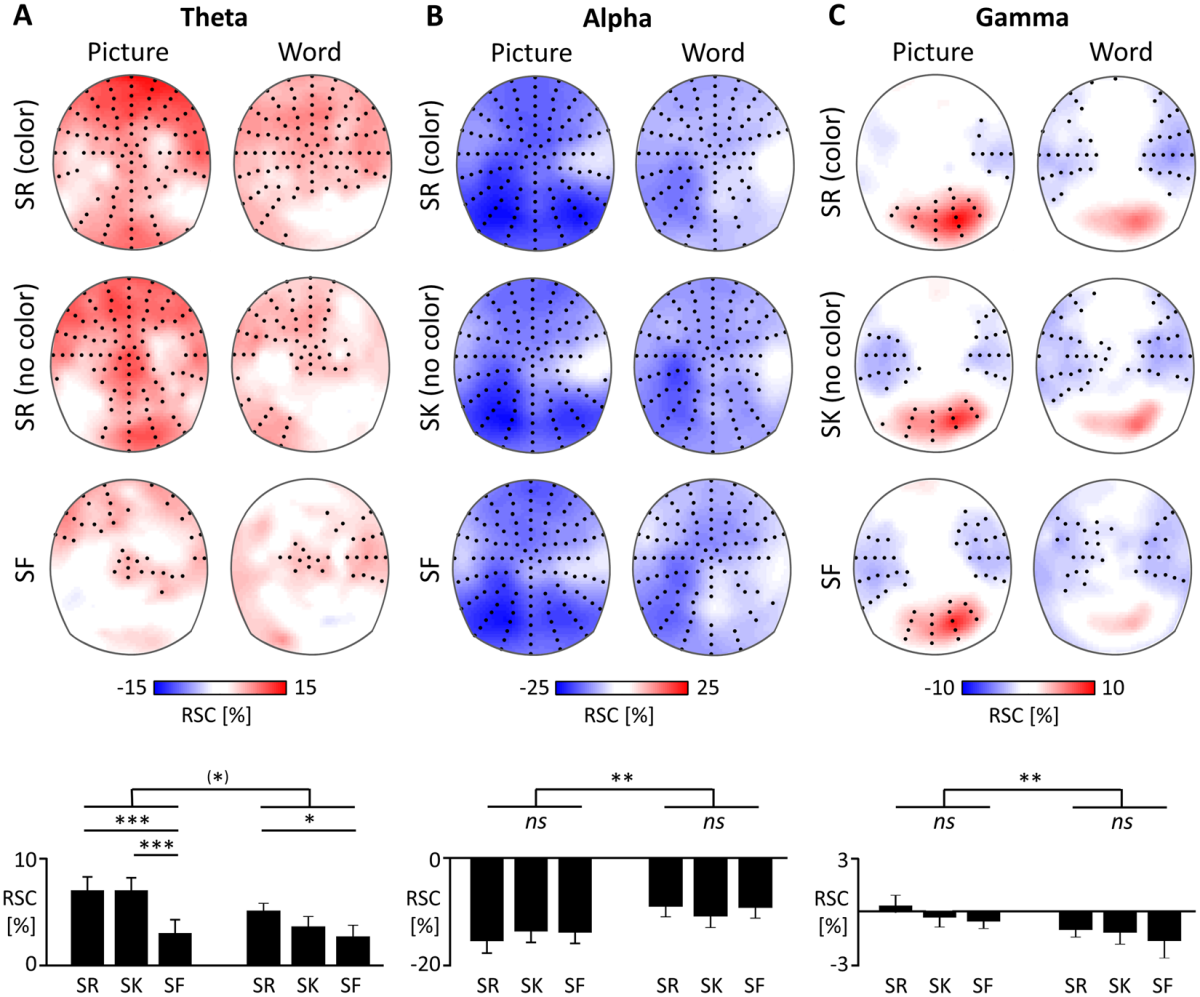
Topographical maps of the encoding activity, in relative signal changes (RSC). Figures indicate the RSCs for subsequently remembered item-color associations (SR, with color), items later retrieved without (SK) and subsequently forgotten items (SF). Topographies show the averaged power for the whole duration of the stimulus presentation (500 ms to 2000 ms) at individual theta, alpha and gamma frequencies. Black dots indicate the electrodes of statistically significant clusters (*p* < 0.05), tested against zero. Bars illustrate the RSCs across all clusters. The main effects of stimulus type and the post hoc t-tests between encoding conditions are displayed, *p = 0.055, *p < 0.05, **p < 0.01, ***p < 0.001.

The spectral changes across participants are displayed in Fig. 3. To test significant increases and decreases in the individual theta, alpha and gamma rhythm, we used a cluster permutation test against zero across all electrodes. All significant increases and decreases are displayed in Fig. 4. Subsequently, we used ANOVAs to test for global differences in theta, alpha, and gamma power across all 9 clusters, see Fig. 4, lower panels. For theta, we found a main effect Response, *F*(2, 50) = 7.67, *p* < 0.001, *η²* = 0.24, with higher theta power for remembered and known stimuli, compared to forgotten stimuli, see Fig. 4A. Furthermore, theta power was marginally higher for pictures compared to words, *F*(1, 25) = 3.23, *p* = 0.084, *η²* = 0.11, for the main effect of the Stimulus type and there was a marginal Stimulus x Response interaction, *F*(2, 50) = 2.94, *p* = 0.062, *η²* = 0.11. Specifically, the theta rhythm peaked at frontal electrodes, pronounced for remembered stimuli and for pictures. This was reflected in a main effect Cluster *F*(8, 200) = 5.70, *p* < 0.001, *η²* = 0.19, as well as a Cluster × Stimulus, *F*(8, 200) = 5.92, *p* < 0.001, *η²* = 0.19, and a Cluster × Response interaction, *F*(16, 400) = 1.91, *p* = 0.029, *η²* = 0.07. A subsidiary ANOVA at frontal clusters revealed a main effect for Response, *F*(2, 50) = 9.65, *p* < 0.001, *η²* = 0.28, and Stimulus, *F*(1, 25) = 7.57, *p* = 0.011, *η²* = 0.23.

In the alpha rhythm we found a stronger decrease in alpha power for pictures compared to words, *F*(1, 25) = 8.95, *p* = 0.006, *η²* = 0.26, main effect Stimulus. Alpha suppression was highest at posterior recording sites for the processing of pictorial stimuli, see Fig. 4B. This is reflected in a main effect Cluster, *F*(8, 200) = 79.40, *p* < 0.001, *η²* = 0.27, and a Cluster × Stimulus Interaction, *F*(8, 200) = 7.74, *p* < 0.001, *η²* = 0.24, and a subsidiary ANOVA at posterior clusters, main effect Stimulus, *F*(1, 25) = 17.61, *p* < 0.001, *η²* = 0.41. There was no difference between subsequent memory conditions, main effect Response, *F*(2, 50) = 0.34, *p* = 0.714, *η²* = 0.01.

For the gamma rhythm, we found pronounced posterior gamma activity over posterior recording sites and reduced gamma activity at temporal electrodes, see Fig. 4C, main effect Cluster: *F*(8, 200) = 13.92, *p* < 0.001, *η²* = 0.35. We found higher gamma activity for pictures compared to words, *F*(1, 25) = 9.09, *p* = 0.006, *η²* = 0.27, but no difference in gamma activity between remembered and forgotten stimuli, *F*(2, 50) = 2.12, *p* = 0.145, *η²* = 0.08. The stimulus effect was somewhat pronounced at posterior electrode clusters, confirmed by a marginal Cluster × Stimulus Interaction, *F*(8, 200) = 1.93, *p* = 0.071, *η²* = 0.07, and subsidiary ANOVA at posterior electrodes, main effect Stimulus: *F*(1, 25) = 15.09, *p* < 0.001, *η²* = 0.38. Posterior electrodes also revealed a trend for a Response effect, *F*(1, 25) = 3.11, *p* = 0.061, *η²* = 0.11.

Given that half of the stimuli were tested immediately and some were tested after a delay, we checked if there were any differences in spectral activity between these stimuli by adding the factor Retention Interval (immediate or delayed) as an additional factor in the ANOVAs. This did not reveal any main or interaction effects for stimuli tested immediately or after about 3 h, all *p* > 0.08, for 24 main effects and interaction across all 3 ANOVAs.

#### Theta-gamma phase-amplitude coupling

**Figure 5:**
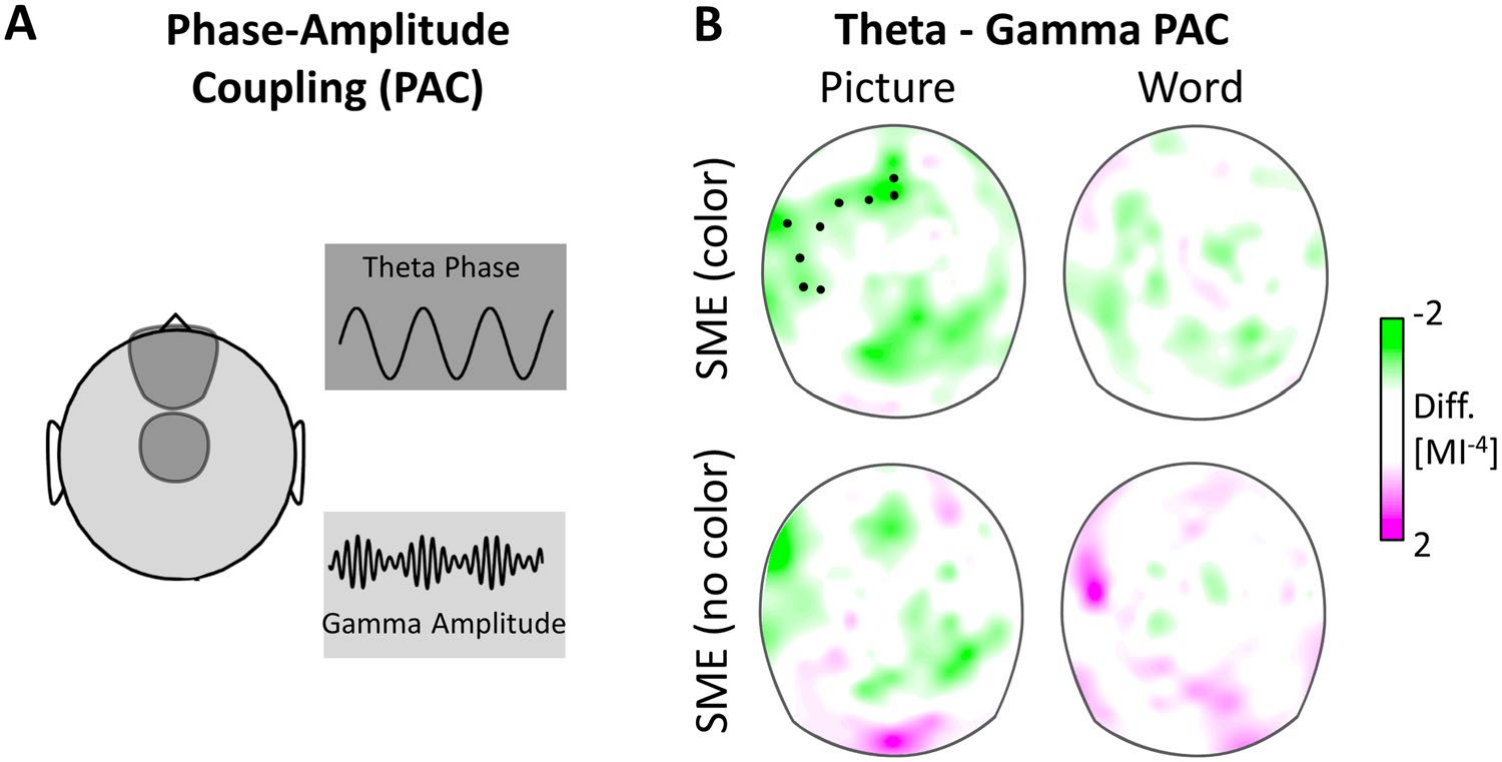
Subsequent memory effects (SMEs) in theta-gamma phase-amplitude coupling (PAC) for items remembered with or without color. (**A**) The cartoon head illustrates theta-gamma PAC, as assessed by the modulation index (MI). Theta-gamma PAC was assessed between individual theta phase across the mid-frontal and -central clusters with the 50–80 Hz gamma amplitude at each electrode. (**B**) Black dots indicate the electrodes of statistically significant clusters (*p* = 0.009), with higher theta-gamma PAC for pictures remembered with color. No theta-gamma PAC was found for the SME of pictures remembered without color, or the SMEs for remembered words.

In a next step we used the MI to quantify phase-amplitude coupling between the fronto-central theta and posterior alpha phase and the gamma amplitude across all scalp electrodes, see Fig. 5A.

In a first step, we entered the MI values of the 9 clusters from the power analyses into a Response × Stimulus × Cluster ANOVA (see above), we found a trend for a main effect Response, *F*(2,50) = 3.089, *p* = 0.054, η² = 0.11 and a Cluster × Stimulus interaction, *F*(4.263, 106.578) = 3.854, *p* = 0.008, *η²* = 0.13. Furthermore, for alpha-gamma PAC, there was a trend for a Stimulus effect, *F*(1, 25) = 3.493, *p* = 0.073, *η²* = 0.12. Given our specific hypothesis on theta-gamma PAC in associative memory processes and previous findings, that theta-gamma coupling effects were found in sparse electrode or voxel clusters^11,19,28^ (and may thus not be well characterized by an omnibus ANOVA), we further tested SME effects in a cluster based permutation test. Therefore, in a second step, we entered theta-gamma coupling into cluster permutation tests, testing the main SME contrasts namely comparing remembered stimuli with and without color against forgotten items, for pictures and words separately.

The cluster permutation test revealed a higher theta-gamma PAC for remembered associations (pictures with color) compared to forgotten pictures (*p* = 0.009) see Fig. 5B. No difference was found between pictures remembered without color, or memory performance for words, when comparing remembered words (with or without color) against forgotten words (all *p* > 0.12). Furthermore, no clusters were found for the same comparisons on alpha-gamma PAC (all *p* > 0.13), not displayed. Find the cluster statistics for the highest clusters of each SME contrast in Table 2.

**Table 2:** PAC clusters permutation statistics.

PAC	Stim	Contrast	Cluster *t*	Cluster *n*	*p*
Theta-Gamma	Picture	SME (color)	21.4659	9	0.009
		SME (no color)	5.7737	2	0.156
	Word	SME (color)	2.7191	1	0.400
		SME (no color)	−6.9872	3	0.128
Alpha-Gamma	Picture	SME (color)	3.1070	1	0.187
		SME (no color)	4.5188	2	0.131
	Word	SME (color)	2.7589	1	0.239
		SME (no color)	2.6089	1	0.285

Peak cluster statistics for all SME contrasts, calculated on the MI values. Cluster t is the sum of the t-values of neighboring electrodes. Cluster n denotes the number of neighboring electrodes forming the cluster. P-values indicate the permutation statistics for the combined permutation tests, separated for theta-gamma and alpha-gamma contrasts.

Critically, differences in cross-frequency coupling between conditions could be biased by power changes of the phase-modulating frequency. Since theta power was higher in the associative memory condition, estimations of theta phase might be improved in contrast to forgotten items. Consequently, the MI measure could be biased. To rule out this possibility, we applied the same control analysis as a previous study (Friese *et al*.^11^): Single-trial data of each participant were sorted regardless of condition by mean theta power averaged across the selected electrodes. We divided the sorted data into quartiles such that the first quartile (Q1) contained 25% of all trials with lowest theta power, and the forth quartile (Q4) contained 25% of all trials with highest theta power. For each quartile, we derived the MIs between the theta power averaged across fronto-central electrodes and the gamma amplitude at individual posterior electrodes, before averaging the MI values across all electrodes of posterior clusters. Naturally, we found an increase in the mean theta power, *F*(1.062, 26.552) = 41.79, *p* < 0.001, *η²* = 0.63. In contrast, MIs did not differ across quartiles, *F*(3, 75) = 1.465, *p* = 0.23, *η²* = 0.06. Hence, it is unlikely that our finding of increased cross-frequency coupling for remembered picture color associations versus the forgotten condition is due to theta power differences between the conditions.

## Discussion

To our best knowledge, this is the first study to dissociate associative binding from visual processes in a subsequent memory task. Theta oscillations increased for subsequently remembered pictures and words, with and without the associated color. In the theta band, this effect was pronounced at frontal electrodes. Critically, theta-gamma PAC was increased between frontal and fronto-temporal electrodes selectively for picture-color associations, and no theta-gamma PAC was found for pictures remembered without color or for words (remembered with or without color). Furthermore, no SMEs were found in alpha-gamma PAC. Alpha, and gamma oscillations were sensitive to visual perceptual processes, with higher alpha suppression and increased gamma power for pictures compared to words. Differences between pictures and words in alpha and gamma oscillations were pronounced over posterior recording sites. Overall, these findings support our main hypotheses; First, the pivotal role of the frontal theta and theta-gamma PAC in binding visual perceptual features, and, second, that alpha and gamma oscillations reflect the gating and promotion of visual perceptual processes along the visual processing stream.

Noteworthy, in the present study we used more conservative analysis strategies than previously^11,13,25^. This is, we based the analyses on all electrodes, included the whole time window of the stimulus presentation, excluding the evoked response, and focused the analyses on specific individual frequencies. Furthermore, 26 participants were included in the analysis and, considering the complexity of the design, we had high trial numbers in each condition.

The present study provides further evidence for increased theta power during successful encoding. This is in line with several former studies using similar study designs^11,13,17,22,25^ (although recent studies with more variable designs and not based on individual frequencies also found lower theta oscillations to be associated with successful encoding^41–43^). The theta rhythm is proposed to mark mnemonic control processes^1,11^. Specifically the theta rhythm plays a pivotal role in ordering and binding perceptual information, reflected in a theta-gamma neuronal code^26^, with first evidence for a sequencing function in the human brain^28,44^. Using visual brain stimulation at an individual theta pace, recent evidence supports a functional role of the theta-rhythm and theta-gamma coupling processes in the formation of novel memories^16^. Theta activity is strongly associated with memory processes in the prefrontal cortex^13,45^ and the medio-temporal lobe^46,47^, two structures which closely interact in long-term memory processes^48^. Proposedly, theta-gamma PAC may provide an optimal temporal code for long-term potentiation processes in the medial temporal lobe^30^, the core system for episodic memories^49^, to establish a coherent representation of time and space^3^. Noteworthy, we found theta-gamma PAC pattern specifically between frontal and fronto-temporal electrodes in the present study, which may result from an interaction between frontal and medio-temporal networks. The present results replicated previous findings on gamma oscillations, coupled to the frontal^11^ or entrained^16^ theta rhythm during encoding. In addition, we could pinpoint that theta-gamma PAC is specific for the formation of visual perceptual feature binding in an associative memory task, which we did not find for non-associative memories or the encoding of words. Thus, theta-gamma PAC may reflect the binding of visual perceptual elements into coherent memories.

Alpha suppression is a marker of attention processes^50^ and plays a critical role in gating perceptual information in visual cortical networks^23,24^. Alpha oscillations may serve perceptual and mnemonic processes by the promotion of visual perceptual information along the visual hierarchy, reflected in the gamma band, with best evidence from cell recordings in the macaque visual cortex^18,51^. Noteworthy, in the present study we found a sharp distinction in alpha suppression and gamma oscillations between pictorial and written object presentations. In particular, alpha oscillations were not sensitive to the subsequent memory condition. These findings add to recent evidence that the alpha rhythm is rather insensitive with regard to the encoding condition^16^ and that event-related alpha suppression during mnemonic processing is rather associated with semantic and attentional processes accompanying the encoding of visual stimuli^22,52^. Thus, speculatively, subsequent memory effects in alpha suppression found in former studies^11,13,22^ may be due to a activation of existing semantic networks in the visual processing stream, which facilitates later retrieval. Noteworthy, by differentiating pictures versus words, we manipulate the complexity of visual perceptual features, processed in the visual modality. However, we do not presume that word processing requires less ‘perception’ or underlies different computational principles. We rather assume pictures provide richer features to be bound and are thus nicely traceable with the scalp-recorded EEG, in contrast to the concepts activated by words, and which may also explain the better remembrance of pictures, compared to words.

Regarding the pivotal role of theta-gamma PAC in associative memory formation, we assume that the theta-gamma code solves two critical computational problems, by mapping real time events to a faster, neuronal time scale. First, the neatly timed rhythmic activation within and across cell assemblies is a precondition for long term potentiation processes in the MTL^30^, implementing the Hebbian principle that cells which “fire together, wire together”^53^. This is, tetanic inputs at the pre and post synapsis are a prerequisite for LTP processes, and thus the formation of novel associations in MTL networks^9^. Furthermore, the theta-gamma code allows neuronal processes to speed up and advance ahead of time to emulate behavioral outcomes, such as place cells, which are activated before a specific location is actually passed^54^.

To conclude, the present findings are in support of a primary role of the theta-gamma code in human associative memory and support the view that theta and alpha oscillations play distinct roles in memory formation. This is, alpha may gate neuronal processes in semantic cortical networks underlying implicit and explicit processes^24^, while the theta rhythms reflects the explicit mental elaboration of perceptual elements, the computational mechanism for associative feature binding^3^. More generally, the theta-gamma neuronal code may implement a mnemonic sampling loop^6^, integrating novel perceptual elements bit by bit into existing representations to guide current behavior in the light of the present context and goals.
